# Study of Water Sorption in Methacryl-Based Polyhedral Oligomeric Silsesquioxane (POSS) Dental Composites Using Molecular Dynamics Simulations

**DOI:** 10.3390/polym15204161

**Published:** 2023-10-20

**Authors:** Chandra Mouli R. Madhuranthakam, Sudharsan Pandiyan, Omar Chaalal, Ali Elkamel

**Affiliations:** 1 Chemical Engineering Department, Abu Dhabi University, Abu Dhabi 59911, United Arab Emirates; omar.chaalal@adu.ac.ae; 2 Schrödinger India Private Limited, Bengaluru 560098, India; sudharsan.pandiyan@schrodinger.com; 3 Chemical Engineering Department, University of Waterloo, Waterloo, ON N2L 3G5, Canada; aelkamel@uwaterloo.ca; 4 Center for Catalysis and Separations, Khalifa University of Science and Technology, Abu Dhabi 127788, United Arab Emirates

**Keywords:** cluster analysis, diffusion coefficient, methacrylate POSS, molecular simulations, water sorption

## Abstract

Methacrylate-based polyhedral oligomeric silsesquioxane (POSS) is one of the new composites used as a dental resin. Both monofunctional methacryl isobutyl POSS (MIPOSS) and multifunctional methacryl POSS (MAPOSS) are reported to be possible resins that possess the desired properties for using them as dental resins. Our group’s previous comparative study on these two resins showed that the MAPOSS composite has superior mechanical properties compared with the MIPOSS composite. In this article, molecular dynamic simulations (MD simulations) are performed to study the water sorption in these two composites. Water sorption in dental composites can have several effects on the material properties, performance, and longevity of dental restorations. Water sorption in MAPOSS and MIPOSS composites is analyzed by studying the hydrogen bonding, cluster analysis, density projection calculations, and diffusion coefficient calculation of water molecules within the resin matrix. MD simulations results are further used to understand the interaction of water molecules with the resin matrix comprehensively, which governs the composite’s mechanical properties. The water sorption study showed that the MAPOSS composite has less water sorption capacity than the MIPOSS composite. The practical significance of this study is to find properties that affect dental restoration and longevity, which can help in the design of better materials for dental applications.

## 1. Introduction

Among the several composite materials used as dental resins, polyhedral oligomeric silsesquioxanes (POSS)-based polymer materials are gaining more importance due to their improved mechanical, viscoelastic, barrier, and thermal properties. Other attributes of POSS include biocompatibility, hydrophobicity, absolute non-toxicity, and the ability to form porous structures that promote cell growth and bone growth [1]. The usage of POSS in dental resins is still developing, and several studies are being carried out on the effect of functionalization. With the multifunctionalization of POSS, several problems related to polymer shrinkage, water sorption, hydrolysis rate, adhesion, and strength can be overcome. For example, the surface of hydroxyapatite (HA), which is used as a filler in dental composites, is modified with methacrylate-bearing silsesquioxane, which leads to improvement in mechanical strength, stability, and compatibility with organic compounds [2]. Methacrylate polyhedral oligomeric silsesquioxane (methacrylate POSS) introduced into dental resins offers several potential benefits. The integration of methacrylate POSS can significantly boost the mechanical properties of dental composites. It can improve hardness, enhance tensile and flexural strength, and increase fracture toughness. One of the known limitations of dental resins is their polymerization shrinkage. By integrating methacrylate POSS, this shrinkage can be reduced, leading to fewer gaps between the tooth and the filling. This reduces the chances of secondary caries and post-operative sensitivity. Dental composites need to withstand the mechanical stresses of mastication. Methacrylate POSS enhances the wear resistance of these composites, ensuring their durability, especially in high-wear areas of the oral cavity. Adding methacrylate POSS can boost the thermal stability of the composite material, ensuring the resin does not degrade or alter its properties easily under varying temperatures in the oral environment. Methacrylate POSS, given its functionalization with methacrylate groups, is compatible with traditional methacrylate-based dental resins. This ensures homogeneous dispersion in the resin matrix, which is crucial for retaining and enhancing the desired properties of the composite. With proper optimization, methacrylate POSS can aid in producing durable and aesthetically pleasing composites with better color stability and translucency resembling natural teeth. POSS structures in dental composites can potentially reduce water absorption rates, minimizing the detrimental effects of water sorption over time. The potential reduction in the release of unreacted monomers due to the incorporation of methacrylate POSS can enhance the biocompatibility of the dental composite, posing fewer risks to the surrounding oral tissues. The compatibility, photocuring behavior, morphology, and mechanical and shrinking properties of two functionalized POSS, methacryl isobutyl POSS (MIPOSS), which has only one methacrylate functional group, and methacryl POSS (MAPOSS), which has eight methacrylate functional groups, are reported by Wang et al. [3]. Their experimental findings are complemented by Madhuranthakam et al. [4] using molecular dynamics simulations (MD simulations). The main polymer matrix in these two resins consisted of bisphenol A glycerolate dimethacrylate (BisGMA) and tri(ethylene glycol) dimethacrylate (TEGDMA). The composition of these resins was varied by using different amounts of POSS, and its effect on the macroproperties asuch as mechanical strength and volume shrinkage was studied [3]. Madhuranthakam et al. [4] found that, as the weight percentage of MAPOSS in the composite increased, the density of the composite increased. In contrast, the density of the composite decreased when more MIPOSS was added. Regarding stiffness, measured by Young’s modulus within the elastic limit from stress–strain data, it was found to rise with the addition of 5 wt% MAPOSS but then it declined. Conversely, the Young’s modulus consistently decreased as more MIPOSS was added. MAPOSS’s double bonds were observed to contribute to polymer crosslinking, thus integrating into the polymer matrix. In contrast, MIPOSS molecules existed independently within the polymer matrix, forming clusters of various sizes, negatively affecting the material properties compared with the control and MAPOSS composites. These observations were validated by Madhuranthakam et al. [4] through density mapping and powder diffraction studies, and were consistent with other experimental findings reported in existing research. It was observed that, as the weight percentage of MAPOSS increased up to 5% in the resin, the Young’s modulus increased, while it decreased when the weight percentage of MIPOSS in the resin increased. It was observed that the MAPOSS resin has more beneficial properties compared with the MIPOSS resin [3,4]. For example, the flexural strength, fracture energy, hardness, and scratch resistance were reported to be better in the MAPOSS resin. In addition to these properties, water sorption in these resins is an important property to be assessed and analyzed. This article mainly focuses on studying the penetration of water molecules through the MAPOSS and MIPOSS dental resins using MD simulations. Water sorption can lead to the softening, swelling, and hydrolytic degradation of the composite matrix and filler particles, reducing mechanical properties such as flexural strength, compressive strength, and fatigue resistance [5]. Water sorption in dental resins leads to filler–matrix debonding and hydrolytic degradation of the filler. Water uptake usually occurs in the resin matrix and constitutes a diffusion-controlled process. The rate of diffusion and degree of swelling of the resin depends on the hydrophilic or hydrophobic nature of the monomers used in the matrix. A matrix with hydrophilic monomers will absorb more water and swell greater than resins made of hydrophobic monomers. Color change in dental composites can occur due to water sorption, which affects the refractive index of the resin matrix and filler particles. Over time, this can lead to a less aesthetically pleasing appearance [6]. Water sorption can cause the composite to swell and create micro gaps at the interface between the tooth and the restoration. This can lead to bacterial infiltration and an increased risk of secondary caries [7]. Further, the swelling of dental composites caused by water sorption can lead to dimensional changes, which may affect the overall fit and performance of the restoration [8]. There are several factors such as curing conversion, composition of the matrix, filler content and particle size, aging, and degradation that can affect the water sorption in dental resins [9,10,11,12,13]. Water sorption in MAPOSS and MIPOSS resins can be understood using MD simulations [14,15], which can provide valuable insights into the interactions, structure, and properties of materials, which can be challenging to investigate experimentally.

In this study, hydrogen bonding, the diffusion coefficient, and density projection analysis are used to analyze water sorption quantitatively and qualitatively. Hydrogen bonding plays a crucial role in water sorption in polymers. Hydrogen bonds form between the water molecules and the functional groups present in the material, leading to water absorption or adsorption. Hydrogen bonding can contribute to the swelling of materials when water molecules form hydrogen bonds with the polymer network, leading to an increase in the material’s volume. This swelling can have significant effects on the mechanical properties, such as reduced tensile strength and modulus of elasticity [16]. On the other hand, the diffusion coefficient of water in dental resins is an important parameter for understanding water sorption. The diffusion coefficient of water in dental resins can be influenced by factors such as the resin matrix composition, filler content, chemical nature of the filler, degree of conversion, and environmental conditions [17]. The rate at which water molecules move within a material is indicated by its diffusion coefficient. This measure is vital for comprehending and forecasting how water interacts with dental composites. When water enters the composite, it can cause the material to expand, altering its size and shape. If the diffusion coefficient is high, water will be absorbed more quickly, leading to rapid expansion. Such expansion can produce tension at the boundary between the tooth and the composite, possibly resulting in detachment or the formation of small gaps. The resin structure and its bond with fillers can degrade due to water, and a quicker diffusion amplifies this effect. The physical strength attributes of dental composites can diminish upon water intake, and this change’s pace may be associated with the diffusion coefficient. Some dental composites have monomers that have not reacted, which may leach out when water is absorbed. A greater diffusion rate can hasten this release, posing a potential oral health risk. The appearance of dental composites can change due to water absorption, possibly causing them to stain. The diffusion coefficient determines the rate and degree of these alterations. The time required for a dental composite to stabilize its water content with its surroundings is tied to the diffusion coefficient, with a higher value indicating quicker stabilization. Density projection analysis (DPA) is another technique employed to investigate water sorption by analyzing the changes in density distribution within the MAPOSS and MIPOSS resins. In the current study of molecular simulations focusing on MAPOSS and MIPOSS resins, it is crucial to grasp how water molecules are distributed or concentrated in specific areas or dimensions. This understanding is facilitated by density projection analysis, where water molecules’ quantity (either number or mass density) is calculated based on one or more spatial aspects. This method converts the system’s 3D spatial data into a more straightforward 1D or 2D format, streamlining the analysis and offering a clearer view of the system components’ distribution and activity. Though DPA is more commonly used for porous materials, it is used in this study to complement the results obtained from other methods. Though the MAPOSS and MIPOSS composites studied here are not liquid materials, the goal is to understand the behavior of water molecules inside these composite pores. Hence, this method is adopted to understand the distribution of water molecules inside the polymer composite matrix.

## 2. Simulations Details

All molecular dynamics simulations are performed using the Materials Science (MS) Suite version 4.8.134 of Schrödinger 2022-4 release (Schrödinger, LLC, New York, NY, USA), which uses the OPLS4 force field [18]. The chemical structures of BisGMA, TEGDMA, MAPOSS, and MIPOSS are drawn using the 2D and 3D sketchers in MS Maestro. Using the Disordered System builder within the framework of MS Suite, desired composite structures are made, which is followed by material relaxation that consists of 20 ps NVT Brownian minimization at 10 K, a 20 ps NPT Brownian minimization at 100 K, a 100 ps NPT equilibration at 300 K, and, finally, 10 ns equilibration at 300 K and 1.01325 bar. The simulation protocol involves using a simulation time step of 2.0 fs with a Nose–Hoover thermostat, MTK barostat, and trajectory recording interval of 5 ps at a temperature of 300 K. Using the exact composition shown in Table 1, crosslinked structures of 5 wt% MAPOSS and 5 wt% MIPOSS with BisGMA and TEGDMA are obtained. More detailed information on the methodology can be obtained from Madhuranthakam et al. [4].

Further, using the Penetrant Loading within the framework of MS Suite, water molecules corresponding to 100% humidity (corresponding to a vapor pressure of 3.535 kPa) are loaded randomly to the MAPOSS and MIPOSS dental resins. No additional water molecules are added above the saturation level. The human mouth’s relative humidity is often high, especially around the 95–100% range, due to the presence of saliva. Thus, when simulating the performance of dental resins, maintaining a high relative humidity can mimic the conditions in the oral cavity. Humidity can influence the polymerization of dental resins. It may affect the degree of conversion, leading to different mechanical properties. The water gradient is altered in less humid conditions, leading to decreased water diffusion into the composite. Lower humidity means fewer water molecules can penetrate the composite material, reducing the diffusion coefficient. In a practical scenario, since dental composites are always exposed to saliva, 100% humidity is justifiable and used in the simulations. Finally, these structures are equilibrated using the material relaxation protocol, and MD simulations are performed for 100 ns NPT at 300 K and 1.01325 bar. The equilibrated systems thus obtained are used for conducting diffusion coefficient calculations, cluster analysis, density projection analysis, hydrogen bonding, and interaction energy calculations.

## 3. Results and Discussion

**Figure 1:**
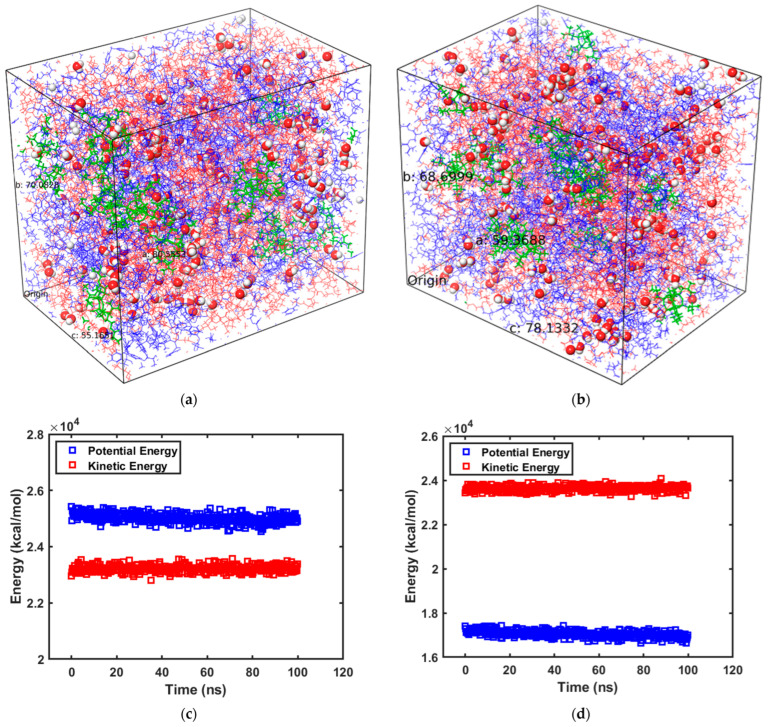
Equilibrated systems of (**a**) MAPOSS and water molecules, (**b**) MIPOSS and water molecules, (**c**) energy vs. time profile for MAPOSS–water system, and (**d**) energy vs. time profile for MIPOSS–water system (blue chains—BisGMA, red chains—TEGDMA, green structures—POSS, red and white spheres are water molecules).

Figure 1a shows the equilibrated crosslinked MAPOSS system with water molecules while Figure 1b shows the corresponding system with MIPOSS (the exact composition of these systems is given in Table 1). Figure 1c,d show the potential energy (which is the energy associated with the positions of atoms or molecules relative to one another) and kinetic energy (which relates to the motion of particles) profiles for MAPOSS and MIPOSS, respectively, for a production run of 100 ns. Figure 1c,d clearly show that the systems reached equilibrium from the overall steady kinetic and potential energy profiles. A similar trend was also obtained for the density of the system, which led to the confirmation of an equilibrated system within the 100 ns production run. Figure 1c,d also show that, in the MAPOSS system, the potential energy is greater than the kinetic energy, while it was observed to be opposite in the MIPOSS system. The slightly higher kinetic energy in the MIPOSS system compared with the MAPOSS system suggests that diffusion will be greater in the MIPOSS system than in the MAPOSS system. Diffusion of water molecules through the MAPOSS and MIPOSS matrices is simulated in detail by running MD simulations at 300 K and 1.10325 bar. Using the linear portion of the mean squared displacement (MSD) curve from the MD simulations, the diffusion coefficient of water molecules in the resins is calculated according to Einstein’s method [19]. The MSD is defined as shown in Equation (1), where $\langle r^{2}\rangle$ is the mean squared displacement over time, *r*(*t*) is the position vector of the diffusing particle at any time *t*, and *r*(0) is the initial position vector

(1) $$\langle r^{2}\rangle =\langle {\left(r\left(t\right)-r\left(0\right)\right)}^{2}\rangle$$

**Figure 2:**
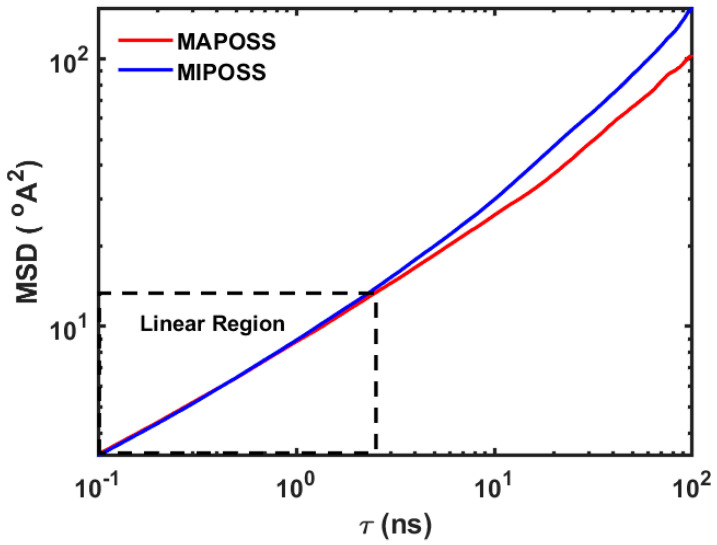
Double logarithmic plot of the MSD vs. time (τ) for MAPOSS and MIPOSS resins.

For a sufficiently long simulation time, the diffusion coefficient, *D*, can be estimated using Equation (2).

(2) $$D=\frac{1}{6N}\underset{t\to \infty }{\lim}\frac{d}{dt}\sum_{i=1}^{N}\langle {\left[r_{i}\left(t\right)-r_{i}(0)\right]}^{2}\rangle$$

where *N* is the number of molecules of that species. The initial portion of the MSD curve where $\langle r^{2}\rangle \;\propto t$ plays a significant role in the estimation of *D* using Einstein’s method. The linearity of this part of the MSD curve has to be confirmed if Einstein’s method for estimating the diffusion coefficient has to be used. This can be confirmed by making a double logarithmic plot of the MSD vs. time [20,21]. Figure 2 shows the double log plot of the MSD vs. time for both the MAPOSS and MIPOSS systems. In this figure, it clearly shows that, up to almost 2 ns, the curve is linear (represented by the dashed rectangular box) and follows the relationship $\langle r^{2}\rangle \;\propto t$ and, after 2 ns, it starts deviating from a straight line, representing a nonlinear relationship. 

For estimating the self-diffusion coefficient of water, a system with 3000 molecules of water is equilibrated and MD simulations are run, from which the corresponding MSD curve is obtained. A summary of the calculated diffusion coefficients is shown in Table 2 for the three systems.

**Figure 3:**
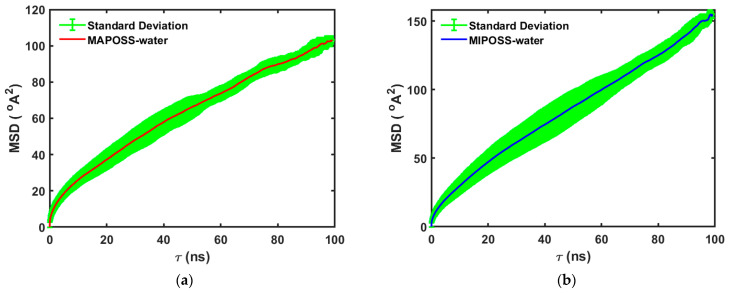
Mean squared displacement (MSD) profiles for (**a**) MAPOSS and (**b**) MIPOSS resins.

The diffusion coefficients obtained for water molecules in both the MAPOSS and MIPOSS systems are in the same range reported for diffusion in other dental resins [5,17,22]. Ferracane [5] discussed the diffusion coefficients of water in various dental resins, reporting values in the range of 10^−12^ to 10^−11^ m^2^/s, consistent with those reported by Sideridou and Achilias [23] in the range of 2.8 × 10^−12^ to 1.1 × 10^−11^ m^2^/s. For the two systems studied in this article, the water diffusion coefficient in MAPOSS is less than the value obtained in MIPOSS resin. The double bonds present in MAPOSS are involved in the curing/crosslinking process, due to which MAPOSS molecules are strongly attached to other polymer molecules in the matrix. But, in MIPOSS, due to the lack of double bonds it acts as a standalone filler and further shows an agglomeration tendency within the polymer matrix [1,2]. The higher diffusion coefficient observed in MIPOSS can be well explained by the uneven free space formed due to agglomeration. Within this space, the water molecules are free to move at a higher speed, which in turn can be observed in the MSD profiles shown in Figure 3a,b. The MSD profiles of water molecules in MAPOSS and MIPOSS resins (see Figure 3) clearly show that the MSD for the MIPOSS system is always greater than the MSD of the MAPOSS system, which supports the observed high diffusion coefficient of water molecules in MIPOSS. 

**Figure 4:**
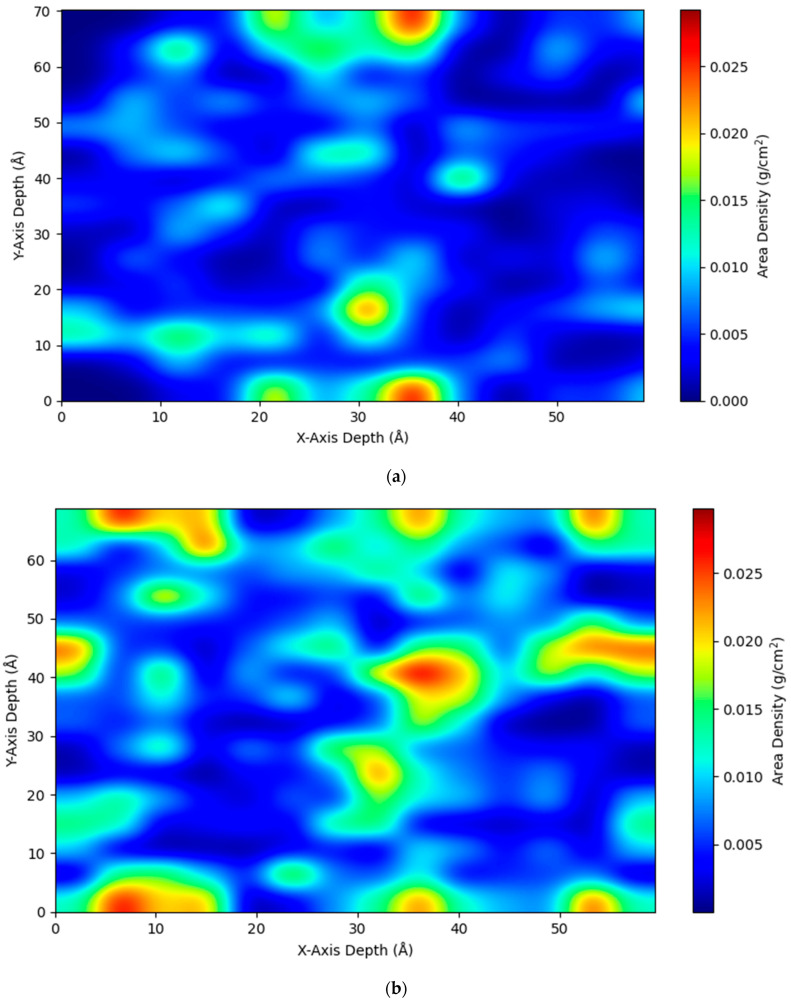
Density projection profiles in the XY direction for (**a**) MAPOSS resin and (**b**) MIPOSS resin.

Further density projection profile analysis, cluster analysis, and number of hydrogen bonds results support the observed water sorption phenomenon in MAPOSS and MIPOSS resins. Figure 4a,b show the density projection profile analysis of water distributed in MAPOSS and MIPOSS resins, respectively. In Figure 4a, water molecules in MAPOSS are more evenly distributed (shown as blue region) compared with Figure 4b, in which water molecules are sparsely distrubuted. The MIPOSS filler in the resin agglomorates, due to which several channels are formed within the polymer matrix through which water molecules diffuse fast (see Figure 4b), which in turn results in a greater MSD value (shown in Figure 2).

In detail, cluster analysis is carried out to study the movement of water molecules through the MAPOSS and MIPOSS dental resins. 

**Figure 5:**
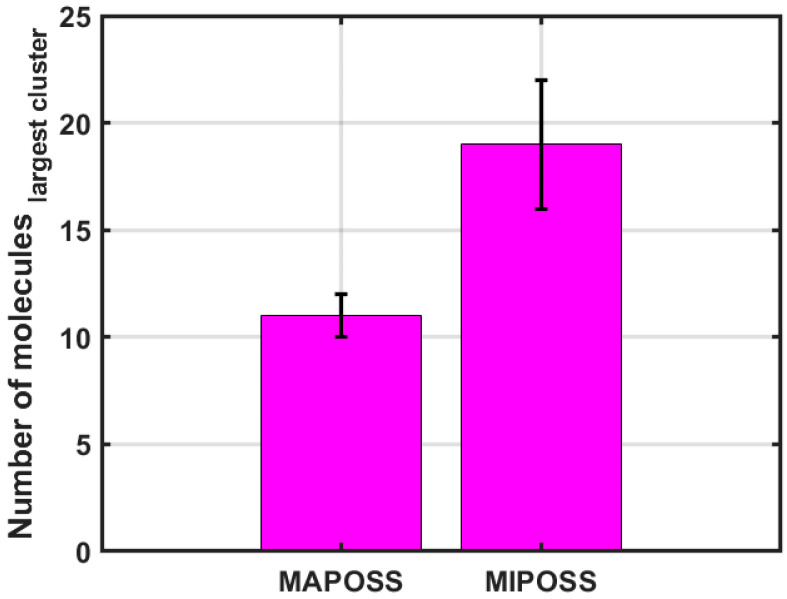
Number of molecules in the largest cluster for MAPOSS and MIPOSS.

Figure 5 shows the comparison of the variation in the number of molecules in the first largest cluster in the MAPOSS and MIPOSS resins, respectively. It is observed that, in the case of MAPOSS, the highest number of water molecules is 11 and an average of 9 water molecules are present for the 100 ns span. The number of molecules within the cluster is found to be steady. But, in the case of MIPOSS, the highest number of water molecules is nineteen, with a highly variable unstable cluster size. After forming a big cluster with 19 water molecules, smaller cluster with fewer molecules are formed. The number of clusters decreases with time in the case of the MAPOSS resin while it increases in the MIPOSS resin. The variation in the mass-weighted radius of gyration is also found to be higher in MIPOSS compared with MAPOSS. From the results, it is found that a greater number of clusters that are unstable are formed in the MIPOSS resin while a smaller number of stable clusters are formed in the MAPOSS resin. This supports the results reported by Madhuranthakam et al. [4] where the agglomeration of MIPOSS molecules causes more uneven space with the resin, which leads to the formation of different size clusters of water molecules, which break and form new clusters during their movement within the channels. A hydrogen bond involves the attraction between a hydrogen atom, which is covalently bonded, and another electronegative atom. In this research study, a hydrogen bond is characterized by a hydrogen-acceptor distance of less than 2.8 Å, a donor angle not less than 120°, and an acceptor angle of at least 90°. The quantity of hydrogen bonds directly or indirectly influences the mechanical properties. Both the MAPOSS and MIPOSS resins have organic matrices with carbonyl and hydroxyl groups that can participate during hydrogen bonding. The presence of hydrogen bonding sites within the matrix can influence the uptake and interaction of water with the material. An analysis of the number of hydrogen bonds in both the MAPOSS and MIPOSS resins shows that the MIPOSS resin has almost the same number of hydrogen bonds as the MAPOSS resin (see Table 3). The number of hydrogen bonds in the polymer–water system is obtained using Equation (3).

(3) $$N_{H,p-w}=N_{H,T}-N_{H,p-p}-N_{H,w-w}$$

In Equation (3), *N_H,T_*, *N_H,p-w_*, *N_H,p-p_*, and *N_H,w-w_* denote the number of hydrogen bonds in the total, polymer–water, polymer–polymer, and water–water systems, respectively. 

**Figure 6:**
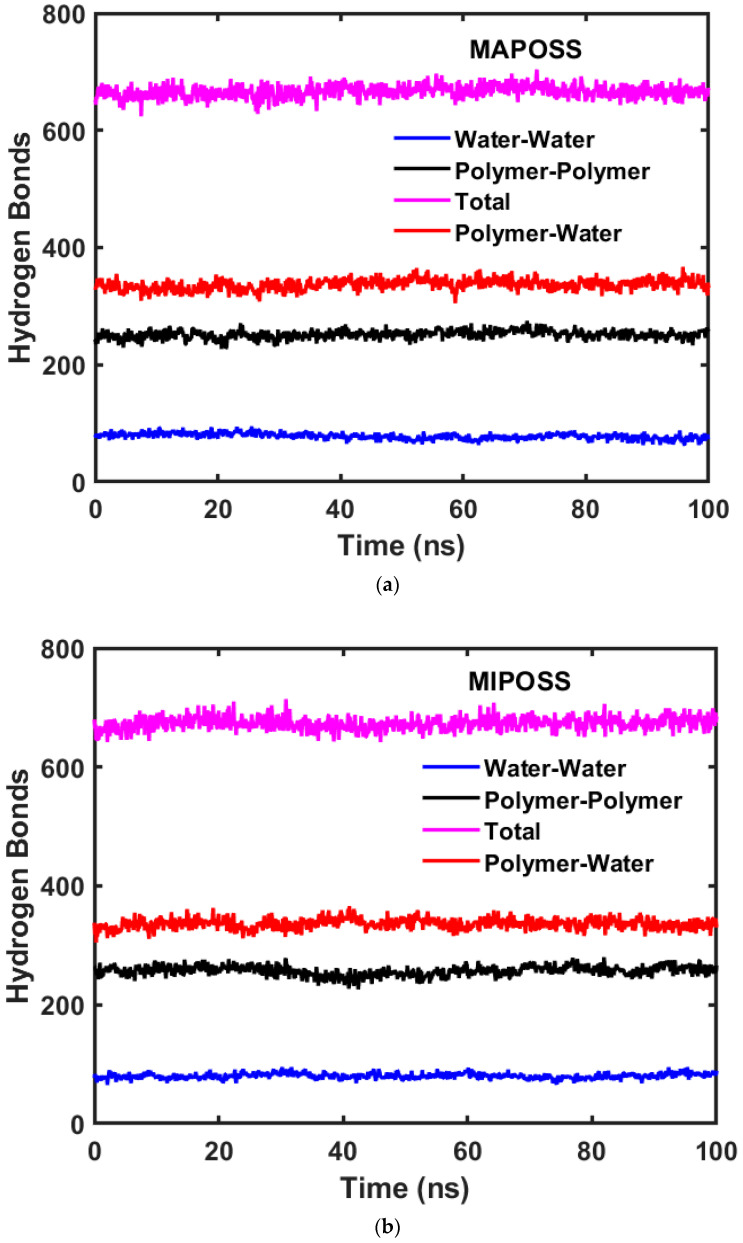
Number of hydrogen bond profiles in (**a**) MAPOSS resin and (**b**) MIPOSS resin.

The number of hydrogen bonds significantly affects the material properties, swelling, and sorption capacity of a material [24], and, in particular, dental resins [25,26]. Figure 6a,b show the complete profiles for the number of hydrogen bonds obtained for the MAPOSS and MIPOSS resins, respectively. Though the number of hydrogen bonds for the polymer–water interactions in both MAPOSS and MIPOSS is almost equal for the same saturation, the diffusion coefficient of water in MIPOSS is observed to be greater than that in MAPOSS. This shows that water molecules can easily penetrate and reach the hydrogen bonding sites through MIPOSS compared with MAPOSS. Hence, though the loading and interactions are the same, the behavior of the resins is different. Experimental data related to mechanical properties such as flexural strength, flexural modulus, wear resistance, and volume shrinkage are reported for MAPOSS and MIPOSS [3]. However, the diffusion coefficient of water in these composites is yet to be determined by conducting experiments. Imazto et al. [27] found that the diffusion coefficient in the polymer made of BisGMA and TEGDMA alone was found to be 1.71 × 10^−12^ m^2^/s, corresponding to 68% degree of conversion, while He et al. [28] reported it to be 0.75 × 10^−12^ m^2^/s for 85% degree of conversion (with 5% water sorption). For the parent polymer, the experimental diffusion coefficient was reported to be 1.6 to 1.7 × 10^−12^ m^2^/s (with different degrees of conversion, temperature, water sorption capacity) [27,28,29]. The diffusion coefficient obtained for MAPOSS resin in this study is closer to this range though the conversion used in the current study is 100% and for a relative humidity of 100%. In general, the diffusion coefficient strongly depends on the degree of conversion, water sorption, and hydrophilicity of the polymer. On the other hand, the sorption of water in the parent polymer matrix (BisGMA and TEGDMA) along with other fillers (such as bisphenol A ethoxylated dimethacrylate, urethan dimethacrylate, ethoxylated bisphenol A dimethacrylate) was studied, and higher diffusion coefficients compared with the MAPOSS and MIPOSS resins were reported [30,31,32]. 

## 4. Conclusions

Sorption of water molecules in nanocomposites of BisGMA, TEGDMA, MAPOSS, and MIPOSS were analyzed using molecular dynamics simulations. These crosslinked resins with a 5 wt% of the filler were used to study the diffusion of water molecules through the polymer matrix. Wang et al. [3] and Madhuranthakam et al. [4] showed that MAPOSS resin outperforms MIPOSS resin. In this follow-up study, it was observed that MAPOSS resin has less sorption of water molecules compared with MIPOSS resin. It was observed that water molecules in the MIPOSS resin had a higher MSD than those in the MAPOSS resin, which completely agrees with the higher diffusion coefficient of water molecules in the MIPOSS resin. The diffusion coefficient of water molecules was 1.7931 × 10^−12^ m^2^/s for the MAPOSS resin, while it was 2.5016 × 10^−12^ m^2^/s for the MIPOSS resin. This increase in the diffusion coefficient was also confirmed from the cluster analysis, which showed that bigger, unstable, and higher numbers of clusters were formed in the MIPOSS resin, while smaller, stable, and fewer clusters were formed in the MAPOSS resin. This was further confirmed in the density projection profiles, which showed an even distribution of water molecules in the MAPOSS system and an uneven distribution of water molecules in the MIPOSS system. Hence, MAPOSS resin has less sorption of water molecules compared with MIPOSS resin, which in turn leads to increased longevity, decreased hydrolytic degradation, and less microleakage of dental composites. 

## Figures and Tables

**Table 1:** Composition and individual number of molecules of dental resins with POSS.

Composite	BisGMA (wt%)	TEGDMA (wt%)	POSS (wt%)	BisGMA (Number of Molecules)	TEGDMA (Number of Molecules)	POSS (Number of Molecules)
MAPOSS	47.5	47.4	5.1	199	357	8
MIPOSS	47.4	47.4	5.2	202	362	13

**Table 2:** Diffusion coefficients of water molecules in MAPOSS, MIPOSS, and water.

System	Diffusion Coefficient (m^2^/s)
MAPOSS—water	1.7931 × 10^−12^ ± 5.3331 × 10^−15^
MIPOSS—water	2.5016 × 10^−12^ ± 9.8694 × 10^−15^
water—water	4.3654 × 10^−9^ ± 2.5966 × 10^−13^

**Table 3:** Comparison of number of hydrogen bonds in MAPOSS and MIPOSS.

Type of Interaction	Number of Hydrogen Bonds	
	MAPOSS	MIPOSS
Water–Water	78 ± 5	80 ± 5
Polymer–Polymer	251 ± 7	257 ± 9
Total	666 ± 11	674 ± 11
Polymer–Water	337 ± 9	336 ± 9

## Data Availability

Not applicable.
